# Heterogeneous Retirement Savings Strategy Selection with Reinforcement Learning

**DOI:** 10.3390/e25070977

**Published:** 2023-06-25

**Authors:** Fatih Ozhamaratli, Paolo Barucca

**Affiliations:** Department of Computer Science, University College London, London WC1E 6BT, UK; p.barucca@ucl.ac.uk

**Keywords:** agent based modelling, retirement finances, deep reinforcement learning, financial computing, portfolio choice, profile heterogeneity

## Abstract

Saving and investment behaviour is crucial for all individuals to guarantee their welfare during work-life and retirement. We introduce a deep reinforcement learning model in which agents learn optimal portfolio allocation and saving strategies suitable for their heterogeneous profiles. The environment is calibrated with occupation- and age-dependent income dynamics. The research focuses on heterogeneous income trajectories dependent on agents’ profiles and incorporates the parameterisation of agents’ behaviours. The model provides a new flexible methodology to estimate lifetime consumption and investment choices for individuals with heterogeneous profiles.

## 1. Introduction

Retirement financing has been experiencing a clear transition trend from defined benefit (DB) schemes to defined contribution (DC) schemes, as reported by [1]. DB schemes require scheme sponsors as ultimate guarantors which can bail out funds in case of deficit. Employers prefer DC schemes because the risk and responsibility of managing funds, longevity risk, and market risks are transferred to contributors in DC schemes. Furthermore, the contribution rates in DC schemes in the UK are on average significantly less, 5.1%, in comparison to DB average contributions of 28.5% [2]. The effects of economic shocks during the accumulation phase are critical; some people were raiding retirement accounts during COVID-19. Under-pensioned groups [3] faced significant wage shocks, and this also affected their future cumulative wealth and earnings. Exceptional government policies were critical to alleviate the effects of COVID-19 on pension savings and wages, but a significant shock with effects to the labour market could not be avoided. It has become apparent how different professions can be affected differently by economic shocks, bringing attention to the role of profile heterogeneity also in the context of pension management. For instance, the rise of the gig economy [4] and irregular workforce participation modes enable more flexible work-life conditions but introduce larger variations to income trajectories due to the lack of guaranteed income streams.

Previous research has addressed the income distribution and its relationship with age [5], which can be used to quantify the effects of demographic shifts and aging population on income. The increasing heterogeneity of career paths and income trajectories require addressing the questions of how much to save in a more consistent way as well as how to allocate the savings between spendable liquid investments and non-liquid retirement investments. The foundations of the theories presented in following section are based on the life-cycle hypothesis of saving by [6], which states that individuals aim to maintain a consistent level of consumption throughout their lifetime. In the literature, the life-cycle models of income, consumption, and portfolio allocation have been analysed from various perspectives. Samuelson approached lifetime portfolio selection [7] in the context of dynamic stochastic programming in discrete time and solved the multi-period generalisation corresponding to lifetime planning of consumption and investment decisions. Merton formulated the continuous-time version [8] of the same approach for portfolio selection under uncertainty. Later, he extended these results [9] to more general utility functions, price behaviour assumptions, and for income generated also from non-capital gains sources. A comprehensive study [10] proposes a life-cycle model of consumption and portfolio choice as a temporal portfolio optimisation problem where labour income is assumed to be a risk-free asset, and where the portfolio choice is calibrated with real-world data. Ref. [10] presents a model where risky income is invested in either risky asset or riskless asset; both are liquid and can be used for consumption, and they model the income process explicitly and analytically. They solve the optimal portfolio allocation problem at a given age by numerical solution of their model with backward induction. A following study by [11] presents a model which includes an explicit formulation of the income process; it differs from previous research by introducing liquidity friction to risky assets by charging an excess cost if consumption is financed through the risky asset. The model must be solved numerically, and the solution is described by authors as slow and difficult due to three continuous state variables, two continuous control variables, and a fixed transaction cost breaking the concavity of the objective function. The Campanale model assumes that a person has the freedom to switch between liquid and non-liquid asset types, which is not the case with locked pension savings. Campanale et al. use dynamic programming to optimise the [12] preference utility of a household, given specific labour income process consisting of the deterministic G(t) of a third-order polynomial and idiosyncratic shock. In the Campanale et al. model, the most important calibration challenge is the transaction cost, which also includes psychological and non-monetary costs.

Further studies focus on liquid and non-liquid retirement savings accounts where liquidity is constrained by introducing cost to liquidate retirement savings [11,13]. Previous research fails to address the heterogeneity of contributor profiles and falls short of addressing the idiosyncratic challenges of avoiding consumption crisis during unemployment periods and saving an adequate pension pot for retirement.

Advances in agent-based modelling of complex financial systems, increased computational power, and advances in techniques for optimising agent behaviour in complex environments motivated our investigation. In particular, a deep learning approach for addressing an economic optimisation problem is introduced in the model called AI Economist by [14]. It uses AI-assisted deep reinforcement learning and implements an agent-based model to address the needs of socioeconomic challenges introduced by designing and testing economic policies, where modules called social planners are trained to discover tax policies in dynamic economies that can effectively trade off economic equality and productivity. A two-level deep reinforcement learning approach is applied to learn dynamic tax policies, based on economic simulations in which both agents and a government learn and adapt.

In this paper, we introduce a simple model of contributor agents who decide how much to save and how to allocate the savings, this decision is affected by state variables, specific behavioural parameters and by the information flow in the peer network. Agents decide and optimise their allocation strategy using a deep neural network trained with reinforcement learning. We introduce a simple simulation environment for the agents, which encapsulates employment and income dynamics. Our research bridges a gap between agent-based modelling of the pension system and deep reinforcement learning for finance.

We provide results from agents trained with a state of the art learning methodology and implementing agent-specific optimal behaviour with high granularity for heterogeneous profiles. The model is dynamic, scalable, and can be calibrated to different scenarios. The results show that the balance between near-term consumption safety and retirement savings can be achieved by profile-specific allocation strategies.

RL algorithms are able to learn from data and adapt to changing conditions that can not be expressed with simple mathematical formulations, which means they can be more flexible and responsive to changes and non-linear dynamics. Our model is suitable for tailoring to specific pension fund management goals and constraints. Our model contributes to development of personalised portfolios, which can factor in profile heterogeneity of age, profession, risk tolerance, and financial goals. The model can be trained to mitigate potential risks such as market volatility, labour risk, and changes to geopolitical conditions as well as sustainability goals. The RL algorithm can be trained to identify and mitigate potential risks that are specific to certain groups of pensioners. All of these can be achieved by incorporating relevant property into simulation dynamics and training the same model with the new simulator. Such a model is also adaptive to changes in the market conditions and can be used for dynamic asset allocation strategies.

The recurrent nature of our deep neural network model makes it possible not only to provide good saving and pension investment decisions at any time given the profile and current data of the agent but also makes it possible to capture historical income trajectory via the recurrent embedding, which is a great difference with available models [8,11,15], where the decisions are made by processing current income but not the trajectory. Our recurrent-neural-network-powered policy model can also learn the dynamics of heterogeneous income trajectories, which is great progress towards more capable decision making of retirement finances.

Our framework is suitable for incorporating extensive behavioural modelling and parameterisation of the agents. It captures the effect of information transmission [16], emphasises consumption sensitivity against negative shocks, as well as covering utility perception [17].

In addition, our model makes it possible for contributors to account for occupation-specific dynamics of life-time income trajectories, which in turn makes it possible to prepare against profile-specific income shocks by allocating savings to cash buffer at the right time frames of their lives.

Our research represents a significant first step to model pension finances in an agent-based model with deep reinforcement learning which permits modelling configurations with increased complexity and realism, in our paper we presented a simple two asset version with simple environment dynamics.

In the following sections, in order to evaluate the performance of the proposed deep reinforcement learning model, we conducted a series of simulations using synthetic data. The simulations were designed to mimic the income trajectories of different occupation groups and to test the ability of the model to determine optimal saving and investment strategies for these scenarios.

We first generated synthetic data for a range of occupation groups, including low-income, medium-income, and high-income groups. The income trajectories for each group were generated using age-dependent income dynamics, with different growth rates and volatility levels for each group. We also included random shocks to the income streams, such as sudden decreases or increases in income due to economic events or changes in employment status.

Next, we used the proposed deep reinforcement learning model to train agents belonging to different occupation groups. The agents were trained with the objective of maximizing long-term wealth while taking into account the age-dependent income dynamics and the income shocks. We incorporated behavioural parameters for each agent, such as risk aversion, shock sensitivity, and individuality factors in order to make the model more realistic and to capture the different decision-making styles of individuals. This is the first time this has been performed for a pension ecosystem.

Once the agents were trained, we ran a series of simulations to evaluate their performance. In each simulation, the agents were given an initial wealth level and were required to make decisions about how much to save and invest in each time period based on their current income and the expected future income. We measured the performance of each agent by tracking their cumulative wealth over time and comparing it to the optimal wealth that could be achieved given the same income streams as well as their ability to sustain themselves during unemployment periods.

Overall, our simulations showed that the proposed deep reinforcement learning model was able to accurately capture the profession and age-dependent income dynamics and that it was able to learn optimal saving and investment strategies for the different occupation groups for the first time. The agents were able to maximise their long-term wealth while taking into account the income volatility, liquidity, and the trade-off between immediate consumption and future savings. These results demonstrate the power of the proposed model for tackling the challenges of personalised retirement planning.

Our model is able to account for the unique income profiles and decision-making styles of each individual, rather than focusing on average or typical income trajectories. This is an important improvement over many previous models by Merton, Campanale, and Cocco, which have often focused on average income trajectories, rather than accounting for the diversity and complexity of individual income profiles.

In terms of empirical results, the proposed model has been extensively tested and validated using simulations calibrated with synthetic data generated from a range of different occupation groups and age ranges. The simulations demonstrated that the model was able to capture the effects of occupation and age on income dynamics, and that it was able to learn optimal saving and investment strategies for the different occupation groups. The agents were able to maximise their long-term wealth while taking into account the potential for income volatility, liquidity, and the trade-off between immediate consumption and future savings towards retirement. These results provide strong evidence that our model is able to provide accurate and effective recommendations for individual saving and investment decisions for retirement finances.

## 2. Model

**Figure 1:**
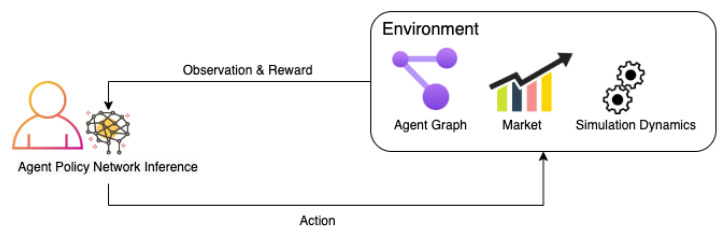
Agent and Environment.

We introduce a simple model where the agents interact with the simulation environment and optimise their savings behaviour. Dynamics of asset prices are features of the simulation environment, and various dynamics can be used, which provides flexibility. For our simulations, we proceed with simple assumptions of constant return rates for each asset class. Endowment dynamics are not hard-coded into the system, and the investment behaviour of our agents at each time step, Figure 1, which is governed by a deep recurrent neural network, determines the agent specific endowment dynamics. These neural networks are trained by reward outcomes from interactions of agents with the environment.

During each cycle, agents observe the environment in which they are situated; they choose to allocate their income between consumption and liquid and non-liquid assets.

Each agent has a heterogeneous profile reflecting the occupation and demographic characteristics; these characteristics are determinants of the unique income and consumption trajectories of each agent. Agents also have characteristic behavioural parameters such as shock sensitivity, consumption utility, and peer-influence factors, which effect the way agents perceive the world and assign value to their stances. In particular, agents are bootstrapped in a social graph which is used for the transmission of information such as employment status.

Each month, agents receive their income according to their employment. Simulated employment and market dynamics, such as asset return rates, are exogenous and provided by the modeller according to empirical observations. The employment dynamics are dependent on heterogeneous profiles (occupation and demography) and include the new employment of unemployed agents.

The agent first decides how much to save and how much to consume, and secondly, the agent allocates the saved amount among a liquid asset and non-liquid asset towards pension savings, each with different return rates. In order to make this financial decision, the agent’s profile, income, behavioural parameters, and peer information observed from their own social network are given as an input to a deep policy network.

Deep reinforcement learning and parallel simulation of nearly 30,000 agents in 100 M timesteps are used for training the deep policy network. The policy network learns an optimal saving and investment strategy for pension savings, avoiding a consumption crisis due to insufficient liquid savings during unemployment.

### 2.1. Optimisation Problem

In the literature, the optimal consumption and investment problem has been expressed as a Bellman value function of consumption and assets optimised by dynamic programming [11]. Each agent receives an income governed by the simulation’s state transition dynamics, T, and makes a consumption and investment decision according a policy π that results in a perceived reward for the agent that can be formulated as

(1) $$r_{i,t}=u\left(c_{i,t},\eta \right)+\Delta x-\psi \chi \left(c_{i,t}-x_{i,t}^{liquid}\right)-\zeta \chi \left(m-c_{i,t}\right)\left(m-c_{i,t}\right)$$

$u(c_{i,t},\eta )$ denotes the the utility from consumption and Δx denotes the change of wealth at current time step *t*, with respect to t−1. A penalty of ψ for not being able to finance current consumption $c_{i,t}$ with liquid savings $x_{i,t}^{liquid}$ is applied by unit step function χ, which can also be related with the concept of borrowing constraint in the finance literature; in our case such a constraint would be applied as a Lagrangian relaxation.

The agent is penalised by ζ for not being able to consume the minimum consumption amount *m*; the penalty is proportional to consumption deficit, where constant relative risk aversion function(CRRA) defines the utility from consumption [18] with η as degree of non-linearity:

(2) $$u\left(c_{i,t},\eta \right)=crra\left(l,\eta \right)=\left\{\begin{array}{cc} \frac{l^{1-\eta }-1}{1-\eta } & \eta \geq 0,\eta \neq 1\\ \ln(l) & \eta =1 \end{array}\right.$$

Reinforcement learning is reliant on feedback from the environment, and strict rules need to be communicated mostly via the reward signal, which makes penalisation necessary in some cases. If the agent is unemployed or allocated insufficient funds to fulfil minimum consumption required by the modeller, then the liquid funds are used to finance consumption. If the funds are insufficient, a consumption crisis occurs, which impacts rewards negatively with a consumption crisis penalty. If the agent consumes a lesser percentage then it is required to finance at least minimum consumption amount, then there is an invalid action penalty.

We can further augment the rewards with agent specific parameters to augment the effects of negative changes. The negative utility difference is augmented with an agent’s shock perception modifier in order to amplify the negative shocks according to the agent’s behavioural parameter κ.

(3) $$\begin{array}{c} f\left(\Delta ,\kappa \right)=\left\{\begin{array}{cc} 1 & \mathrm{if}\;\Delta \geq 0\\ e^{\kappa } & \mathrm{if}\;\Delta <0 \end{array}\right. \end{array}$$

which can be used as a function of the reward excluding penalties. The updated reward can be defined as

(4) $$r_{i,t}^{shaped_{1}}=f\left(u\left(c_{i,t},\eta \right)+\Delta x,\kappa \right)-\psi \chi \left(c_{i,t}-x_{i,t}^{liquid}\right)-\zeta \chi \left(m-c_{i,t}\right)\left(m-c_{i,t}\right)$$

We can shape the reward to incorporate additional relaxed constraints to improve training stability of the neural networks, and one such modification can be applied to the penalty of the consumption decision leading to consumption insufficiency; we should penalise the agent only if the current income is exceeding the minimum consumption amount, which means we do not penalise the policy network π for something that it is not in control of because the simulation T is in control of the income. The updated formula can be defined as

(5) $$r_{i,t}^{shaped_{2}}=u\left(c_{i,t},\eta \right)+\Delta x-\psi \chi \left(c_{i,t}-x_{i,t}^{liquid}\right)-\zeta \chi \left(m-c_{i,t}\right)\left(m-c_{i,t}\right)\left(E_{i,t}-m\right)$$

Agents try to maximise the discounted rewards that they receive during the simulation:

(6) $$\max_{\theta }E_{a_{i}∼\pi_{\theta },s^{\prime }∼T}\left[\sum_{t=0}^{T}\gamma^{t}r_{i,t}\right]$$

The goal is to maximise the expectation of the γ discounted reward $r_{i,t}$ over the entire epoch of *T* periods, which denotes the entire epoch of *T* months. These rewards are determined according to the income that they obtain, which is determined by the simulation T and their decisions $a_{i,t}$ following the policy π. The state of the environment is updated according to $T(s_{t+1}|s_{t},a_{t})$. Agents maximise their γ discounted expected return for time periods 0 to *T*, which denotes each month, depending on the agent state $s_{i,t}$ and the policy parameter $\theta_{i}$.

(7) $$a_{i}∼\pi_{\theta }:\pi \left(a_{i,t}|s_{i,t},\theta \right)$$

Our policy function π is a deep neural network with weight parameters θ, which obtains the agent specific state $s_{i,t}$ as input.

We are looking to find an optimal parameter $\theta^{*}$ for our policy function π that maximises the expected return of discounted rewards.

(8) $$\theta^{*}=\underset{\theta }{\mathrm{argmax}}E_{a_{i}∼\pi_{\theta },s^{\prime }∼T}\left[\sum_{t=0}^{T}\gamma^{t}r_{i,t}\right]$$

We calibrated the simulation with census data and trained a deep recurrent neural network for policy estimation.

### 2.2. Training the Model with RL

Rewards from the environment are used to make the probabilities outputted by the $\pi_{\left(a_{i,t}|\theta \right)}$ policy function more accurately, and we accomplish this by back-propagating the gradients of the objective function to optimise the θ parameters. Reinforcement learning uses feedback from environment to optimise the weights of the model towards more accurate estimation; it is achieved by defining an objective function to maximise or a loss function to minimise. In this paper, we use a policy optimisation technique. In our case, there are two networks; one is a policy network, and the other is a value network. The value network is used during training of the policy network, and such an architecture is called actor critic models [19]. The policy network is responsible for selecting actions by generating action probabilities, and the value network is used during training to evaluate the goodness of each selected action.

The agents select an action $a_{i,t}$ according to the policy $\pi (a_{i,t}|s_{i,t},\theta )$ at a given state $s_{t}$; these actions are saving and portfolio allocation decisions, and these decisions can result changes in the agents wealth and current consumption. The environment calculates a reward $r_{i,t}$ according to chosen reward functions described in Section 2.1. The rewards $r_{i,t}$ at the end of each time-step are used to calculate the estimated advantages ${\hat{A}}_{t}$ during an entire epoch; these advantages are used to optimise the policy network and the value network $V_{\theta }$. The model is trained with the proximal policy optimisation method during the value function, and it is clipped and advantages are normalised, and a standard stable baselines implementation of the [20] PPO2 algorithm is used, which is based on OpenAI PPO2 Algorithm [21]:

(9) $$L^{CLIP}\left(\theta \right)={\hat{E}}_{t}\left[min(p_{t}\left(\theta \right){\hat{A}}_{t},clip\left(p_{t}\left(\theta \right),1-\epsilon ,1+\epsilon \right){\hat{A}}_{t}\right]$$

where the θ is the policy parameter, ${\hat{E}}_{t}$ denotes the empirical expectation, ϵ is a hyperparameter of the clipped surrogate objective of the actor, and $p_{t}$ is the probability ratio under the new and old actor policies:

(10) $$p_{t}\left(\theta \right)=\frac{\pi_{\theta }\left(a_{t}|s_{t}\right)}{\pi_{\theta_{old}}\left(a_{t}|s_{t}\right)}$$

Advantage estimations ${\hat{A}}_{t}$ are calculated with truncated version of generalised advantage estimation (GAE) [22] for *T* timesteps, where $V(s_{t})$ is value function of the critic, and $r_{t}$ denotes reward at time-step *t*, and γ denotes the discount factor:

(11) $${\hat{A}}_{t}=\delta_{t}+\left(\gamma \lambda \right)\delta_{t+1}+...+{\left(\gamma \lambda \right)}^{T-t+1}\delta_{T-1}$$

(12) $$\delta_{t}=r_{t}+\gamma V_{\theta_{t}}\left(s_{t+1}\right)-V_{\theta_{t}}\left(s_{t}\right)$$

where for bootstrapping

(13) $$V_{\theta_{t}}\left(s_{t=0}\right)=0$$

Value function of the critic is clipped with same ϵ hyperparameter of the actor to constitute loss function that is minimised [23] where $V_{target}$ is the sum of advantage and value:

(14) $$L^{V}\left(\theta \right)=max\left[{\left(V_{\theta_{t}}-V_{target_{t}}\right)}^{2},{\left(clip\left(V_{\theta_{t}},V_{\theta_{t-1}}-\epsilon ,V_{\theta_{t-1}}+\epsilon \right)-V_{target_{t}}\right)}^{2}\right]$$

as

(15) $$V_{target_{t}}={\hat{A}}_{t}+V_{\theta_{t}}\left(S_{t}\right)$$

The composite objective function constitutes the actors clipped surrogate objective function, the clipped squared error loss of the critic’s value function, and *S* an entropy bonus as described in [21]:

(16) $$L^{CLIP+V+S}\left(\theta \right)={\hat{E}}_{t}\left[L_{t}^{CLIP}\left(\theta \right)-c_{1}L_{t}^{V}\left(\theta \right)+c_{2}S\left[\pi_{\theta }\right]\left(s_{t}\right)\right]$$

where entropy [24] is defined over action probabilities for *n* actions given a state as

(17) $$S\left[\pi \right]\left(s\right)=-\sum_{i=1}^{n}\pi \left(a_{i}\right)log_{e}\pi \left(a_{i}|s\right)$$

Each epoch is simulated, and the advantage estimations are calculated the model is trained with the composite objective function and stochastic gradient updates with Adam optimiser [25].

### 2.3. Agent and Environment Cycle

In order for the simulation to be integrated with existing frameworks, the AEC (agent environment cycle) [26] is followed to also provide a standardised GYM-Like API. The simulations are vectorised and run in parallel. For the purpose of this research, the simulations are conducted in parallel utilising 32 processors, where each processor runs a cohort of more than a thousand agents. For each time step, all of the agents observe and act simultaneously.

Agents observe the environment; these observations include information regarding the market, graph, and agent’s own state, including occupation, age, income, and wealth.

The agent action $a_{i,t}$ is shaped by policy $\pi_{i}$ during learning of the reward $r_{i,t}$ for the agent and is the sum of total discounted utility and penalty for consumption crisis, which denotes the situation where the agent cannot finance its consumption $c_{i,t}$ governed by consumption dynamic *C*.

The actions are percentage choices between consumption and savings and investment choices between pension orientated non-liquid funds and liquid funds that can be used at any time to finance consumption; these funds have a vital function especially during the times of unemployment.

Agent behaviour is shaped by influences from peers, individuality factors, consumption utility, and shock response characteristics.

The agent policies are modelled with a deep neural network, which takes as input agent-specific observations and a hidden-state:

(18) $$a_{i,t}∼\pi_{\theta }:\pi \left(a_{i,t}|s_{i,t}=\left(o_{i,t}^{network},o_{i,t}^{agent},o_{i,t}^{market}\right),h_{i,t},\theta \right)$$

The parameter variable θ is not agent specific but common for all contributor agents, and the hidden state is updated during action inference of policy network, where the state $s_{i,t}$ constitutes observations of the agent:

- $o_{i,t}^{network}$: Observation of the network.
- $o_{i,t}^{agent}$: Observation of own behavioural factors, income, and resources.
- $o_{i,t}^{market}$: Observation of the market.
- $h_{i,t}$: Hidden state. The updating of hidden state can be interpreted as agents updating their risk profile given observations and previous state, and in the future, the hidden-state can be used as risk profile embedding.

The action space is as follows:

- $a_{i,t}^{save}$: Decides to save x% (and consuming (100−x)%).
- $a_{i,t}^{liquid}$: Decide to allocate y% to liquid asset x% (and allocating the non-liquid asset (100−x)%).
- Saving and liquidity percentages are discretised into bins such as [0,0.25,0.5,0.75,1] in the model.

The full list of variables can be found in the Appendix A.

### 2.4. Deep Policy Network for Optimal Saving, Investment, and Liquidity

Agent observations are expressed as a single vector that comprises the concatenation of agent, market, and graph vectors. The observation vector is passed through the deep neural network towards the LSTM [27], which updates the agent’s hidden state and outputs a vector for next layer, which is softmaxed to output a vector representing the action probabilities. A single policy network is trained for all the actions, and the action can be as follows: “(’C25’, ’L75’)”, where “C25” means consume 25% and save 75%; “L75” means allocate 75% of your savings to liquid assets and 25% of your saving to non-liquid assets.

**Figure 2:**
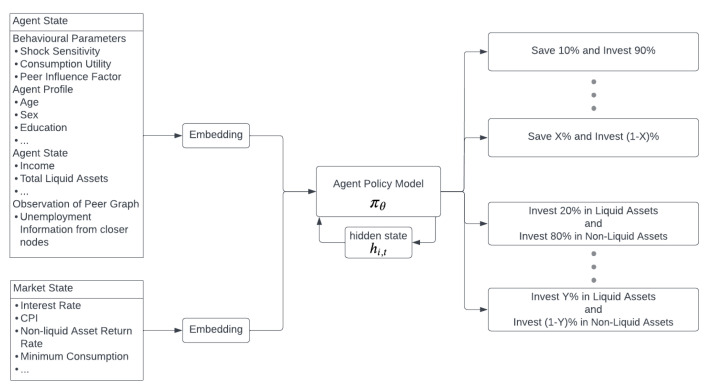
Policy Model.

The hidden states from the model can be thought of as risk profile embedding, which is updated by observations and processing the agent profile with the observed environment and shocks via a deep neural architecture that can be found on Figure 2. Reinforcement Learning is used for adjusting the allocation profile according to the risk profile embedding also expressed as a hidden state. At each time step, the agent decides to allocate the income among consumption, savings, and investment classes. This is accomplished by a deep neural network constituted of several layers of a feedforward neural network and an LSTM, which is responsible for acting as the memory of the agents.

The details of the neural architecture can be found in the Appendix B.

There is a single action space unifying the choices of consumption and liquidity preference, which means that there are not two different networks for different decisions but one unified network which represents the collection of actions such as “(’C25’, ’L75’)”. Setting the reward function for the agents is arguably the trickiest part of the training process; different reward function structures can give spurious and unintended conclusions, which makes the hyper-parameter tuning for the penalties paramount. Failing to tune the penalties results in unintended shortcuts that obstruct the main goal of optimising agent behaviour in an understandable and meaningful way.

After retirement, agents do not act according to their policy networks but according to the desired retirement pension target such as 80% of labour income being pension income or receiving a constant pension amount. These time-steps are still used for advantage estimation calculation that spans all of the epoch and for training the hidden-state evolution weights of the LSTM, which means after training, although we do not use the policy output of the LSTM, we do train the hidden state update weights.

### 2.5. Behavioural Parameters of Agents

For modelling behaviour, we base our parameterisation on the approach in [28], where the authors investigated the applicability of the theoretical domains framework outside clinical uses for cross-disciplinary implementation and other research on behaviour change and provided a simplified version containing 14 domains and 84 component constructs. The theoretical domains framework includes many factors and reports on pension behaviour tend to focus on few factors; for the scope of our research, we chose three factors:

- Consumption Utility: How do they value current consumption? An agent-specific consumption utility multiplier factor
- Shock Response Characteristics: How do they respond to the shock? A factor reflecting how sharp do agents react to the shock and how drastic are they decreasing their consumption.
- Individuality Factors: How are they being affected by each others beliefs and decisions.

In our simulations, each agent has constant risk-aversion parameter $\eta_{i}$ that is randomly assigned at the beginning, but our model allows the risk-aversion to vary during simulation and being fed as input to decision module. Variations of the risk-aversion parameter could be used to capture external effects to risk aversion, which are not captured by simulation captured profile properties such as age, profession, wealth, etc. Some agents are optimistic and underestimate the severity of the shocks, and some agents are pessimists and overestimate the effect of the shocks. The shock sensitivity factor $\kappa_{i,t}$ is a multiplier of the perceived shock effect, which is normalised for agents of the same occupation. It can be assigned from a normal distribution, can be controlled for experimentation, or fed from empirical report. The agents are affected by the peers and the shocks experienced (if $z_{i,t-1}=0$). The observations are informative for the closer agents on the graph and becomes less informative for other agents with weaker connection on the graph. The shocks that affect agents are also weighted with the shock-sensitivity parameter. In our simulations peer effects are limited to observation of a shock propagating through peer network, which provides a signal to adjust their own behaviour well before the shock potentially effects the agent; in the presented simulations, only the peer effects of income groups of low, mid, and high are captured. In this paper, the shocks are not in focus, so the graph structure is simplistic and changes in the income are only governed by age and profession. In more complex simulations, we can use the peer observation to adjust the agent’s own behaviour well before a shock, such as disease, automation, or supply chain shocks (whose propagation can be represented on a graph) potentially reaches the agent. The behaviour parameters that are introduced in this section are kept fixed during the entire simulation.

## 3. The Environment

At each time step, the environment operations are executed first. Agent environment operations are executed as follows: first, the market dynamics is executed, which ensures that assets are gaining value according to the calculated interest rates determined by the modeller. Secondly, essential population dynamics are executed such as ageing of agents, and agents are removed from the system according to the age-specific death probability. The retirement process checks if any new agents are required to retire due to age. If an agent retires, their retirement pension is calculated as a rate of their previous consumption during employment according to the recommended guidelines of the OECD, which refers to ideal pension income being 80% of labour income, which is used as initial pension income. An alternative that is investigated is having a constant pension income such as minimum consumption amount. If an agent is retired, then the agent collects pension from a non-liquid pension fund that they contributed to during employment life. The agents that are not retired are processed to determine stochastically if they will lose their employment and, if so, for how long they will stay unemployed according to the unemployment duration distribution dependent on the occupation and age. Unemployed agents are assigned new incomes at their new jobs according to the income distribution depending on occupation and age. These distributions are fed as quantiled distribution tables to simulation. The employed agents receive their salary each month according to their predetermined income.

The agents decide how to allocate their income between consuming and saving and decide to allocate the saved amount in liquid and riskless assets or non-liquid and low-risk assets. The decision is shaped by learnt policy, observations which include the market dynamics, information regarding actions, and information from peers, and considering the agent’s own profile. We aim to demonstrate the capability of the model to capture long-horizon decisions such as investing in illiquid pension funds. Our model is flexible to broaden the asset classes to include risky but high-return assets such as stocks, but for our demonstration, we wanted to focus on the decision of individuals to allocate the income to pension savings that is unreachable by individuals until retirement but known to have robust returns due to professional and diversified management. The other asset that is captured is liquidity, where it is known to have only minor return but is necessary to finance immediate needs such as periods of unemployment. The focus in not optimal asset allocation of a fund among assets but the investment decision of a person into pension funds or liquidity.

For simulations we made a narrow assumption based on a very small return rate to liquid assets and a small but larger return rate to non-liquid assets which can be assumed as pension fund investments. The model allows agents to be trained for different asset return rates, but the focus is on profile heterogeneity and not asset return rates, so the training assumed asset return rates fixed with the parameters are reflected on the model card.

### 3.1. The Graph and Synthetic Population

A synthetic representative population is used for the initialising agent population, and information such as age, income, profession, education level, and other relevant background information are included.

We assume the employee network consists of three communities divided by income level as low, medium, high, and the three communities have significant intracommunity interaction but limited intercommunity interaction. The graph choice is based on the idea that geographical and social networks are also characterised by the socioeconomic clusters, and the choice of three communities with income levels is the simplification of the socioeconomic network. The synthetic database is generated according to the basic insights from the surveys. Later investigation could incorporate survey data to bootstrap the population and investigate geographical graph, potential social network data, and known network structures to model connections between agents.

Observation of graphs can be done in several ways; a simplistic way is modelling information transmission between each agent and its vicinity, i.e., the first and second neighbours, including transmission of employment information. A more advanced graph observation might be modelled as transmission of not just employment information but also incorporating additional information such as occupation and the income or consumption data; moreover, the near-neighbour graph can be represented with state-of-the-art graph embedding methodology. $A_{a,b}$ is 1 if there is an edge a-> b and 0 if there is no edge between two agents of indices a and b, and δ(x,y) is the Kronecker delta $E_{i,t}$, which denotes the current earnings, ι individuality factor. We can formulate a simple information transmission from the immediate vicinity of neighbours and their neighbours as

(19) $$o_{a,t}^{network}=\sum_{b}\left[\sum_{c}A_{b,c}\delta \left(E_{c,t},0\right)\right]+\sum_{b}A_{a,b}\delta \left(E_{b,t},0\right)$$

Observations from network are augmented with the agent specific individuality factor, simplest case is using the individuality factor as a multiplier to the observation:

(20) $$o_{a,t}^{network\_perceived}=o_{a,t}^{network}*\iota_{a}$$

For the purpose of experimentation and investigation of the model, a synthetic but representative population can provide both fidelity and flexibility in a controlled environment. As a design choice for the synthetic population network, we include three clusters, which can be thought of as three neighbourhoods; these neighbourhoods possess nodes with three different income groups: high, medium, and low income. Each node is connected to its own neighbourhood node, and the neighbourhoods are connected to each other with specified weights. Agents are bootstrapped with one of the general occupation groups, occupation-specific incomes, employment status, and ages derived from US Census Data [29]. Census data are used to generate the synthetic agent population.

### 3.2. Simulation Processes

The simulation is initialised by bootstrapping the agent population and processes. During each time step, the simulation dynamics such as obtaining income and getting employed if unemployed are applied first, and then the agent decides to allocate income for the consumption or saving and decides to save by investing in liquid assets, which can be liquidised easily during unemployment, or non-liquid assets, which are towards a future retirement but usually have better return. Agents are bound by constraints such as the need to consume a minimum amount determined in light of government statistics [30] that determine a minimum consumption per individual.

The occupation-specific income for new employment is determined according to the summary tables from US Census Data. The tables reflect the quantile breakdown, and the agents are probabilistically assigned to one of the income quantiles.

The unemployment events and employment processes are explicitly modelled and calibrated with the US Census Data [29]. The probability of unemployment and the duration of unemployment are determined according to the summary tables of the US Census.

Retirement age and retirement income can be accounted for in the system. For the sake of simplicity, initial simulations neglect the retirement period, by only focusing contribution period, but the system is later extended to cover the retirement period. Retirement income is defined as a fraction of the last income; fractional retirement income is recommended by international institutions, and this methodology is often also used in the literature [10].

The agent death probabilities are modelled using the Actuarial Life Table [31] in order to make the model comparable with existing models in the literature.

### 3.3. Scaling

The agent observations are continuously scaled and standardised, with an online methodology. This is due to the fact that the training dataset is generated continuously during simulation and the distribution of the observed dataset is not known in advance at the start of the simulation, but it can be learnt to an extent after several epochs, and these learnt scales can be utilised in the following training and inference as well. The relevant agent variables(“OCC_CODE”, “income”, “consumption_utility_factor”, “shock_sensitivity_factor”, “individuality_factor”, “non_liquid_asset”, and “liquid_asset”) are transformed to a vector by concatenating categorical hot vectors with the values of the continuous variables; here, the standardisation of these categorical variables is challenging due to the variability of quantities such as accumulated liquid assets. Huge differences in value may introduce instability during the training of the machine learning models. The market state captures important variables such as interest rates given to different asset classes as a dictionary, and the market dictionary is transformed to a vector as well.

## 4. Results

**Figure 3:**
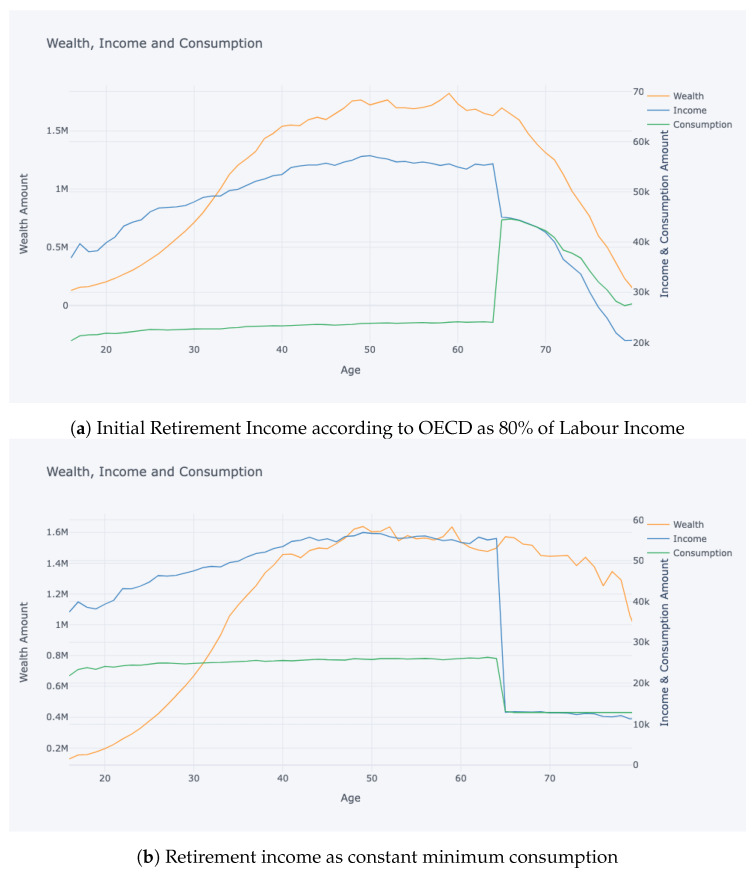
Wealth, consumption and labour income vs. age plot.

**Figure 4:**
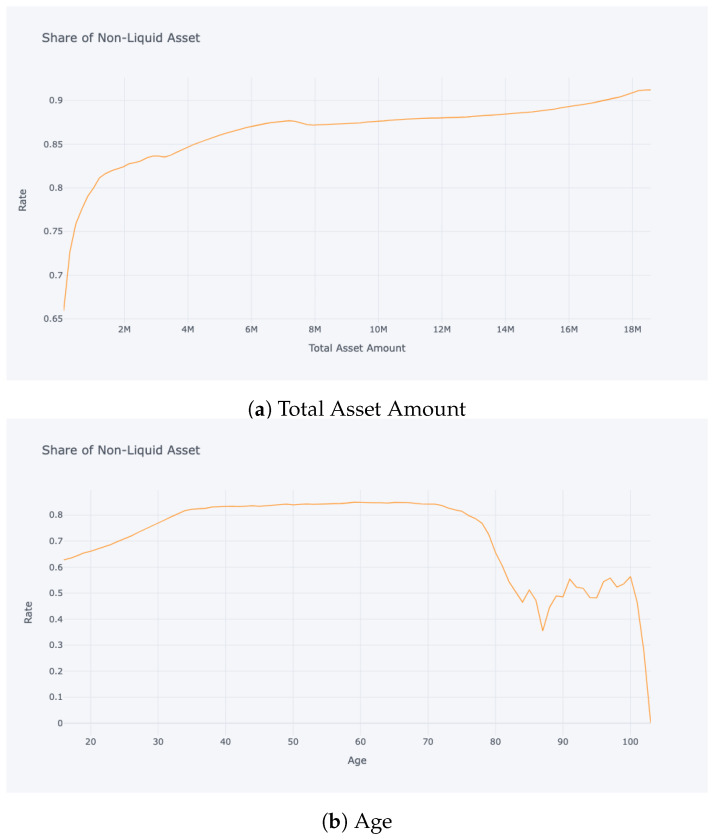
Non-liquid asset share vs. total asset amount and age.

In this study, we adopted a robust approach to gauge the quality of the model fit within the RL paradigm. During training, the accumulated rewards served as an intrinsic metric to track the agent’s progress. Specifically, a steady uptick in rewards over iterations is a positive indication of the agent mastering its interactions with the environment. Post-training, our evaluation focused on contrasting stylised facts derived from the simulated data with empirical evidence and established literature. Stylised facts refer to characteristic patterns and properties that align with real-world observations. Figure 3 and Figure 4 are particularly noteworthy, where trajectories depicting wealth, consumption, and labour income with respect to age, as well as the non-liquid asset share concerning total asset amount and age, show a striking resemblance to the findings of [10]. Additionally, aggregate statistics presented in Table 1, such as occupation and age versus the share of non-liquid investments for wealth quartiles, were compared with results from [11] with high transaction costs (TC). These comparisons are critical in determining the model’s capability to accurately replicate the inherent dynamics of the real-world system.

We look at longitudinal trajectory plots and strategy breakdown per total asset size, which provide granular information regarding the differences between occupations. These plots can capture various scenarios such as differences between early career and mid-career saving rate strategies among various occupations, which provides more tailored strategies for short-term consumption security and healthy long-term pension finances. Twenty-two initially identical parallel cohorts are simulated for 1000 weeks of agent-time in order to generate the resulting tables and plots, which results in 40 M agent time-step samples.

### 4.1. Labour, Income, Consumption, and Wealth

Figure 3 reflects a similar shape of average simulated income, consumption, and wealth accumulation and decrease over the life cycle compared to [10]. The simulated income trajectory is a reflection of the observed data, which is used for calibration of the environment, and the shape of decrease by retirement age is due to the retirement income being defined as a fraction of last income, which then gradually decreases. The consumption trajectory during the work-life reflects saving choices of the population. The agent saves during work-life for financing potential unemployment periods and for retirement finances. The pension income and consumption at retirement age of 65 converges to the determined retirement income percentage of 80% of latest salary. The data becomes noisy for older ages of 80, which might be due to significantly smaller sample size.

The rewards of agents during the simulation can be decomposed to two periods; the first period is the labour participation part, where agent works and gets an income according to income dynamics. In this period, the policy inference module $\pi_{\theta }$ will make decisions of consumption and portfolio allocation and obtain a reward as a result of the current and previous actions; these rewards are used for determining the advantages for training the model. The second period is the retirement period, where the agents make decisions by the pre-determined conditions of the modeller, and these pre-determined conditions can have a constant pension or a pension denoting a certain percentage of labour income. The consumption decision is pre-determined, and there is no portfolio allocation decision during retirement; the retirement income and retirement consumption are in other terms hyperparameters or constraints that are given to our model, but during the second period, agents still obtain a reward, which is used for advantage estimation and also for training the recurrent neural network, where the embedding is still updated and the rewards are used to train the RNN.

(21) $$\sum_{t=0}^{T}\gamma^{t}r_{i,t}=\sum_{t=0}^{T\_retire}\gamma^{t}r_{i,t}+\sum_{t=T\_retire}^{T}\gamma^{t}r_{i,t}$$

**Figure 5:**
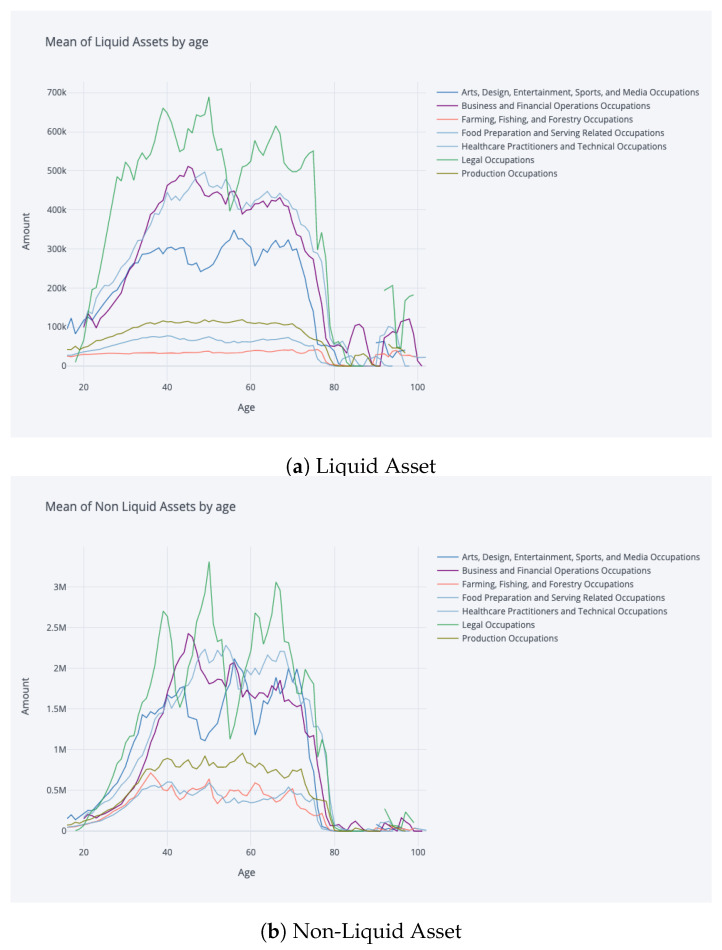
Liquid and asset amounts by occupation at age, where only a selection of occupations are depicted on plot for clear visibility. The different characteristics of occupation groups are reflected by plots.

During retirement, the pension income is supposed to come from pension savings that have been non-liquid during work-life, but if the pension savings are depleted, any liquid savings can be used to finance the retirement income on Figure 5. An interesting outcome of mandating pension income at retirement to be 80% of employment income is comparatively lower consumption during employment, which might not be desirable, but our optimisers were forced into high saving rates due to the 80% mandate, which is stipulated by the literature, and detailed information can be found in previous sections focusing on the literature. One alternative that is investigated is the constant pension income at retirement, where the pensioner obtains a minimum consumption amount as a pension during retirement, which results in much smoother pension savings withdrawal as reflected in Figure 3b. The results indicate that OECD targets are difficult to reach for a significant part of the population.

In Figure 3, we present two contrasting scenarios that depict the consumption patterns of individuals before and after retirement. In Figure 3a, the model is trained with an initial retirement consumption target set at 80% of the final income earned during employment, following the OECD guidelines. This represents a relatively high consumption aspiration upon retirement. The model simulates conservative consumption behaviour throughout the working years, emphasizing saving and investing, in order to meet this substantial retirement target. This is evident from the sharp increase in consumption at the age of 65, which is the transition point from employment to retirement. Conversely, Figure 3b illustrates a scenario where a more lenient retirement consumption target is set. Here, the target is a constant consumption level slightly above the minimum necessary consumption amount. This lower retirement target leads the model to learn a policy wherein consumption during the employment years is markedly higher since a smaller budget is required to meet the retirement consumption goal. There might be various solutions to this problem that are out of the scope of this paper, such as easing pension level mandate, or government contributions, or higher returns of investment. The presented results on profile heterogeneity are based on the simulation conducted in parallel to OECD target of labour income’s 80% as pension income.

### 4.2. Saving Profiles

**Figure 6:**
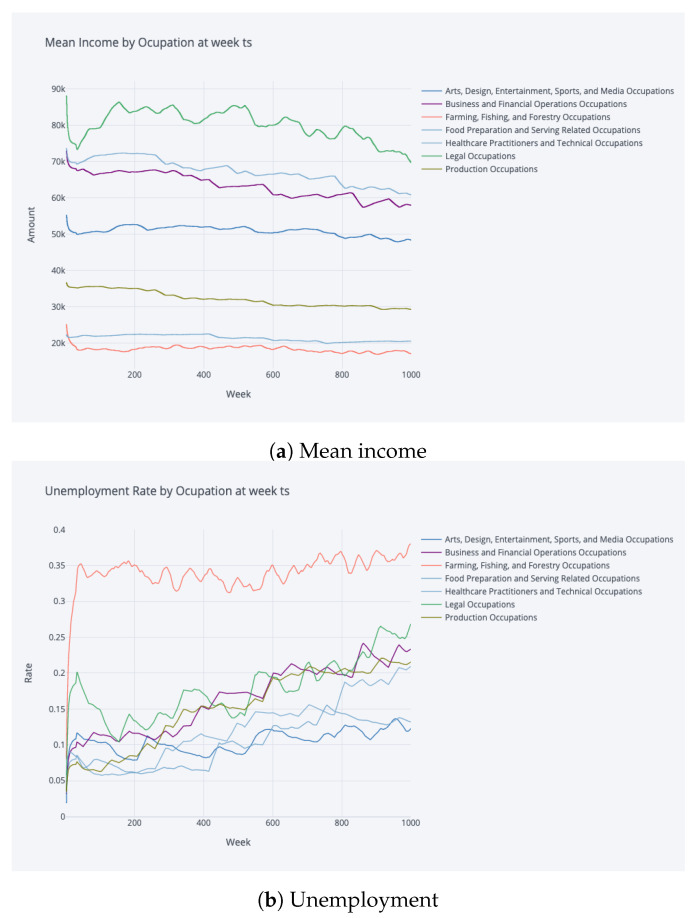
Mean of income and unemployment by occupation at week ts; the values are smoothed by 30-week moving average, and only a selection of occupations are depicted on plot for clear visibility. The different characteristics of occupation groups are reflected by plots.

The evolution of occupational income in a time frame of nearly 20 years in Figure 6 reflects different characteristics for each occupational group, i.e., occupations such as “Sales and Related” and “Transportation and Material Moving” reflect significantly lower mean incomes with lower variance characteristics. On the contrary, occupations such as “Legal” and “Management” reflect the highest mean incomes and high variance of income for each occupation group. This plot reflects even at the simplest level that the income characteristics of each occupation can differ greatly. The unemployment characteristics reflect great diversity, where occupations such as “Farming, Fishing, and Forestry” possess greater and fluctuating risk profiles, which might be partially due to the characteristics of seasonality in these specific occupations. No obvious dependence of saving rate or non-liquid investment rate on age or income level can be found in the analysis, showing the complexity of the decision making happening in the system.

**Figure 7:**
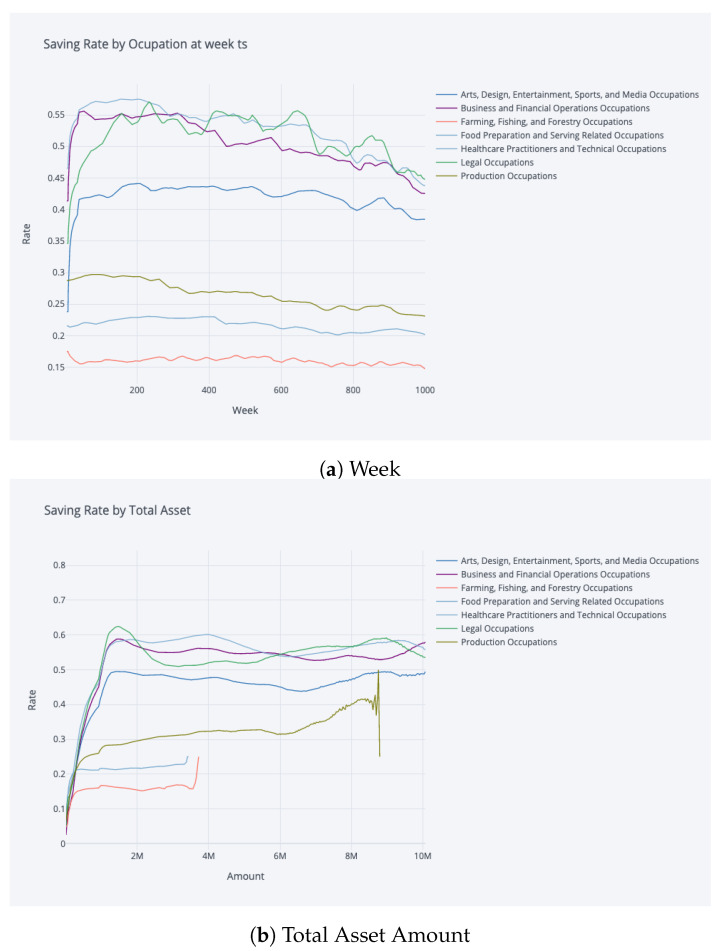
Saving rate by occupation at week ts and saving rate by occupation at amount capped at 10M, the values are smoothed by 30-data-point moving average, and only a selection of occupations are depicted on plot for clear visibility.

The savings profiles in Figure 7 reflect heterogeneous characteristics, where at the same total wealth, the saving rate differs greatly, which can be due to different income levels and unemployment risks of occupations. The saving rate plot shows increasing noise at higher wealth levels near 10M and a much clearer trajectory at lower wealth. An interesting insight is that at the lowest wealth levels, all occupations display similar saving rates. The minimum consumption requirement has a direct consequence of lower saving rates by occupations with low incomes such as “Farming, Fishing, and Forestry”, “Building and Grounds Cleaning and Maintenance”, “Personal Care and Services”, and “Food Preparation and Serving Related”, which have very low saving rates due to their difficulties to finance minimum consumption. Some general patterns can be identified, such as lower income occupations tend to have lower saving rates, but it does not imply that income itself can explain saving decisions; as we can observe, varying saving rates among “Healthcare Practitioners”, “Legal Professionals”, and “Business and Financial Operations”.

### 4.3. Portfolio Allocation

The results of our model are in line with the existing literature on the relationship between the share of non-liquid assets and age distribution. As shown in Figure 4, our model exhibits similar patterns and rates as those found in other studies.

In particular, our model’s results are comparable to those of [11], who also differentiate non-liquid and liquid assets with transaction costs for switching between them. Furthermore, the similarity is particularly strong when the transaction costs are high.

Additionally, our model’s results on the share of non-liquid asset according to total current wealth also reflect a similar shape of an initial increase followed by a plateau. This concurs with the findings of [11].

**Figure 8:**
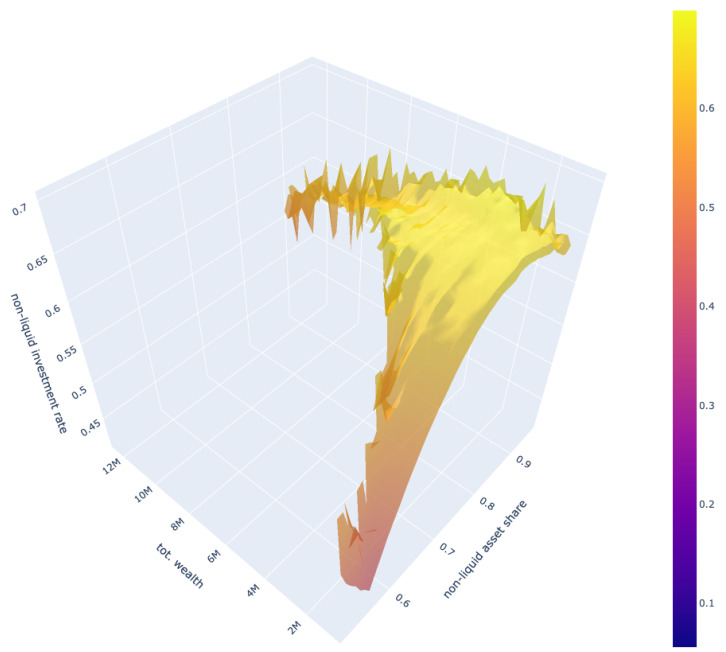
3D surface plot of share of non-liquid assets in x-axis with respect to total asset wealth in y-axis and corresponding decision of non-liquid asset investment rate in z-axis; the values are smoothed with 9-week moving average for clearer visibility.

The relationship between the share of non-liquid assets and age as inferred from our model is consistent with the existing literature as well as the empirical data presented by [11]. Furthermore, the representation of this relationship in our model is further nuanced, as demonstrated in Figure 8, which reflects a more heterogeneous relationship with a greater level of granularity compared to the previous literature.

**Figure A9:**
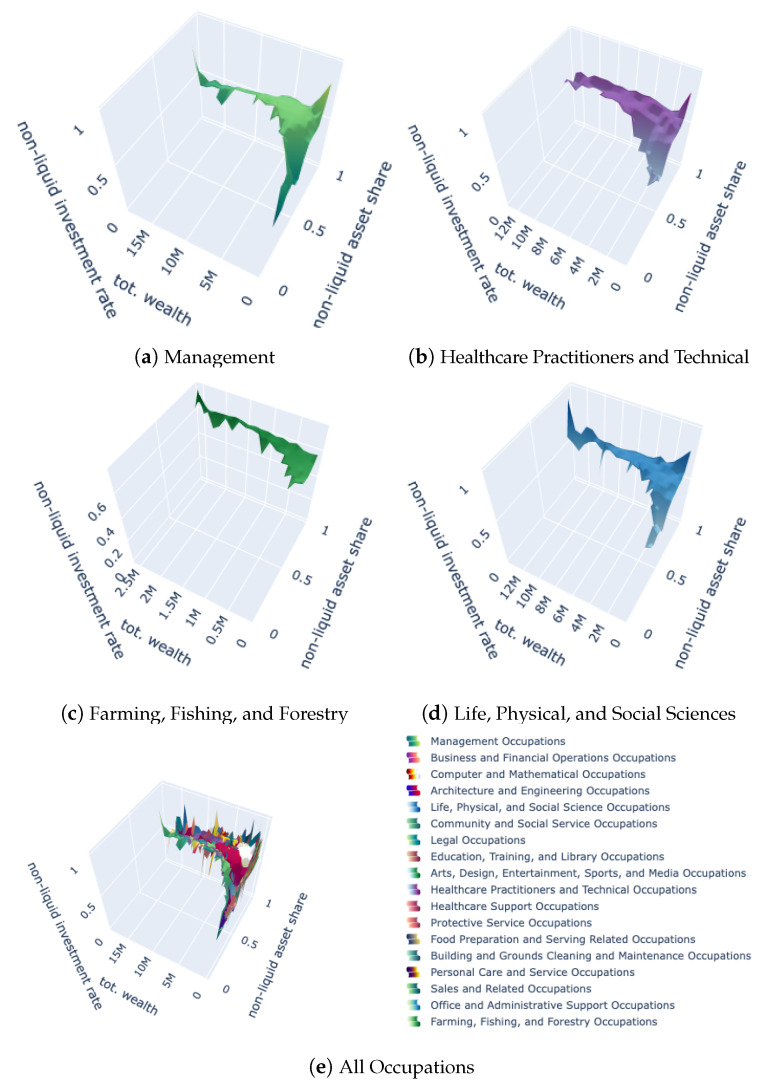
Occupation-specific 3D surface plots of share of non-liquid assets in x-axis with respect to total asset wealth in y-axis and corresponding decision of non-liquid asset investment rate in z-axis; the values are smoothed with 9-week moving average for clearer visibility.

The results of this study suggest that consumption and non-liquid investment decisions should not be based solely on total assets at a specific point in time, as is commonly studied in the literature. Instead, our analysis suggests that these decisions should also take into account the unique income trajectories of individuals as determined by their occupation and age. This highlights the importance of incorporating the heterogeneity of individuals and their specific economic conditions into the analysis of consumption and investment decisions. This is reflected in the findings presented in Table A2 and Table A4 and the 3D plot in Figure A9 that is in the Appendix.

Our model provides a comprehensive representation of income, consumption, and wealth dynamics, as well as portfolio allocation strategies that are suitable for a wide range of heterogeneity and income processes. Furthermore, the level of granularity our model provides is higher than most models in the literature, allowing for a more precise understanding of the investment and consumption decisions made by individuals across different demographic groups.

In summary, the results of our model are in line with the existing literature regarding the relationship between the share of non-liquid assets and age. However, our model goes further by providing a more detailed representation of this relationship, which is suitable for a wide range of heterogeneity and income processes. The granularity of our model also allows for a more precise understanding of the investment and consumption decisions made by individuals across different demographic groups.

A limitation of the model is the presence of a high level of noise in the portfolio allocation 3D surface depicted in Figure 8, which may be a result of the increased complexity of the model. The empty areas on the plot indicate that individuals with higher total wealth tend to have a higher share of non-liquid assets in their portfolios, which is likely due to the higher potential returns associated with these assets and the fact that wealthy individuals have a greater amount of cash buffers as liquidity to finance their consumption during periods of unemployment. It is worth noting that a different model that stipulates higher minimum consumption levels for individuals with higher wealth might lead to some changes in the plot, but the plot is consistent with empirical data and the characteristics of the model.

**Figure 9:**
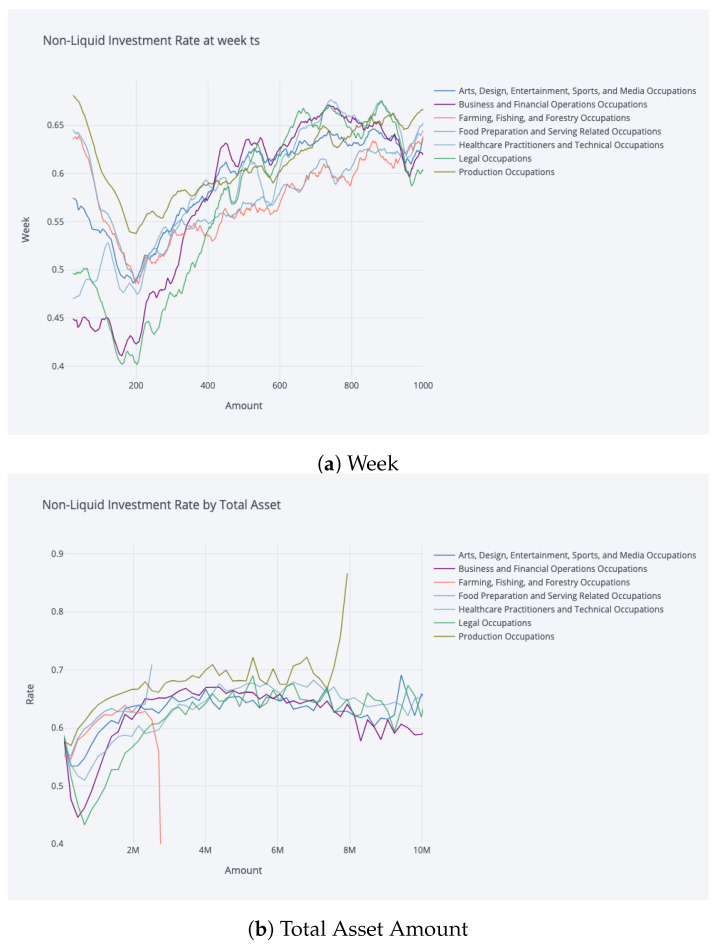
Non-liquid investment rate by occupation at amount capped at 10 M; the values are smoothed by 30-data-point moving average, and only a selection of occupations are depicted on plot for clear visibility.

Contrasting the general saving rate and the non-liquid investment rate characteristics of occupations with respect to total assets results in interesting findings. The non-liquid investment rate by total asset among occupations diverges less than the saving rate by total asset, but still the characteristically differentiating investment strategies are evident in Figure 9. We also observe a noteworthy increase in non-liquid investment rates among production occupations and a stark decrease in farming, fishing, and forestry occupations in relation to total wealth. We believe that these conspicuous shifts, particularly in marginal cases, are likely influenced by outliers present in our dataset. It is conceivable that within lower-income professions such as farming, fishing, and forestry, there exists a small fraction of individuals who have accrued a significant wealth, standing as outliers within their occupational groups. The model’s interpretation of these outliers can be twofold. First, due to the scarcity of training samples representing high wealth within these occupations, the model may extrapolate and learn policies that seem unexpected or non-intuitive, culminating in the steep decline depicted in Figure 9. Alternatively, the model could be capturing genuine characteristics of these outliers, but the limitations in our dataset render us unable to provide a conclusive explanation.

Saving rate by total asset generally increases for all occupations with more assets, with exponential-like increase; then, it plateaus and slightly varies with noise. Saving rates by highest total asset amounts fluctuate greatly, which might be due to different dynamics governing their decisions such as capital income or behavioural parameters weighing more themselves rather than income being the determinant of the decisions.

Our analysis reveals that the proportion of non-liquid investments in relation to total assets is notably higher for individuals in low-income occupations, with the exception being for those with very high levels of total assets where high-income occupations may surpass low-income occupations in terms of non-liquid investment rate. This disparity can be attributed to the fact that low-income individuals have a greater need for liquidity in order to meet short-term consumption needs during periods of unemployment. This finding can be taken into account by policy makers in formulating policies aimed at mitigating risks faced by low-income workers, such as providing unemployment benefits or increasing early-career pension contributions from the government.

Our research makes a significant advance by focusing on the distinction between risky non-liquid savings, such as endowments to defined contribution pension funds, and riskless liquid savings, which can be used to finance immediate consumption. This approach departs from the previous literature in finance, which only focuses on the dichotomy between risky or riskless assets without liquidity constraints.

The distinction between liquid and non-liquid savings offers a more nuanced understanding of consumption and saving decisions made by individuals. It also allows for a detailed examination of how factors such as income and occupation influence these decisions and how they might inform policy design aimed at promoting financial stability for all individuals.

In addition, the focus on the difference between liquid and non-liquid savings offers new insights into how investors evaluate the risk-return trade-off. It takes into account that the risks associated with non-liquid assets may be different from those of liquid assets, which is a crucial departure from standard portfolio optimisation.

Furthermore, this research also aligns with the principle of utility maximisation, where individuals make choices that maximise their satisfaction or happiness. The research highlights how individuals from different occupation groups, income level, and age differ in their choice of investments and the proportion of liquid vs. non-liquid savings. This aligns with the principle that individuals will make choices based on their specific circumstances.

Additionally, in order to account for the potential negative consequences of not being able to finance immediate consumption, our model incorporates penalties for such failures in its analysis. These penalties help to accurately reflect the real-world consequences of not having sufficient liquidity and are an important aspect of the model’s overall representation of the consumption and saving decisions made by individuals. Additionally, we also include the parameterisation of negative income shocks and their effect on the consumption and investment behaviour. This allows us to account for the impact of unexpected events such as job loss or economic downturns on individual financial situations and behaviours.

A comparison of our model’s results with those of Campanale et al. is presented in Table 1 under the assumption of high transaction costs illustrates that our model generally results in a higher proportion of non-liquid investments in total asset portfolios, with some exceptions where Campanale et al. identify a similarly high non-liquid asset share. Furthermore, our analysis highlights the substantial variations in non-liquid asset shares in relation to income quartile and age group, which vary significantly across occupation groups.

The use of a deep reinforcement learning model allows for a more flexible and personalised approach to estimating lifetime consumption and investment choices. Additionally, the focus on heterogeneous income trajectories allows the model to better reflect the diversity of economic conditions experienced by individuals in different occupation groups and at different stages of their lives. The proposed model generates consumption and retirement saving strategies that account for heterogeneous income dynamics specific to an individual’s occupation and age.

## 5. Conclusions

We modelled a pension ecosystem, where heterogeneous contributors make consumption and investment decisions with Deep RL, which advances available models by providing better granularity and accounting for profile heterogeneity.

We provide a novel methodology to optimise agent behaviour for consumption and investment between pension savings and liquid cash buffer, which is flexible and can be calibrated to work in various scenarios and capture agent heterogeneity. Our model does not need an explicit formulation of the income process and can work with empirical data.

Our research represents a first example of end-to-end modelling of pension ecosystems, and it provides a general model to optimise the behaviour for heterogeneous contributors in a dynamic environment. We introduce a single-actor RL model of pension environment, which constitutes a significant step towards multi-actor RL modelling of the pension ecosystem. We successfully devised optimal contributor portfolio allocation strategies between non-liquid pension savings and liquid cash buffers as well as optimal consumption decisions, which can be calibrated with the behavioural parameters of agents. We accomplish this by minimising the consumption crisis periods of agents and maximising the retirement savings.

One of the main limitations of previous work is that it has often relied on simplifying assumptions, such as the assumption of a constant risk-free rate of return or the assumption of a constant level of volatility for all individuals. Another limitation is that previous work has often focused on average or typical income trajectories, rather than accounting for the diversity and complexity of individual income profiles. Finally, previous work has often relied on static optimisation techniques which do not account for the dynamic nature of retirement planning and the potential for changes in income and investment options over time.

One of the key benefits of our deep reinforcement learning model is its ability to simulate different economic scenarios and evaluate the effects on individuals’ saving and investment strategies. This can be useful for policy makers and financial advisers who want to understand how different economic conditions, such as market fluctuations or changes in income levels, can impact individuals’ retirement savings. By simulating these scenarios, our model can provide insights into the potential risks and opportunities that individuals may face, and help them make more informed decisions about how to manage their retirement savings. In addition, our model can be easily adapted to incorporate new data and changes in economic conditions, making it a valuable tool for ongoing analysis and decision making in the field of retirement finance.

The development of models adaptable to diverse policy scenarios, such as varying retirement age regulations and incentive schemes, can require substantial computational resources. The extension of these models to address different sets of government policies is a topic left for future research.

Overall, our simulations showed that the deep reinforcement learning model was able to capture the effects of occupation and age on income dynamics and that it was able to learn optimal saving and investment strategies for the different occupation groups. The agents were able to maximise their long-term wealth while taking into account the income volatility and the trade-off between immediate consumption and future savings. These results demonstrate the value of our model for providing personalised recommendations for individual saving and investment decisions, taking into account the unique income profiles of different occupation groups.

In conclusion, the proposed deep reinforcement learning model is a novel and effective approach for addressing the challenges of retirement planning. By incorporating individual behavioural parameters and using a dynamic optimisation approach, the model is able to capture the unique income profiles and decision-making styles of individuals, providing more personalised and realistic recommendations for saving and investment decisions. The extensive simulations conducted using synthetic data demonstrated that the model was able to capture the effects of occupation and age on income dynamics and to learn optimal saving and investment strategies for different occupation groups. These results provide strong evidence that the proposed model is able to provide accurate and effective recommendations for individual saving and investment decisions. Overall, the proposed model represents an important contribution to the field of retirement planning and has the potential to provide valuable insights and guidance for individuals looking to plan for their retirement.

## Figures and Tables

**Table 1:** Occupation and Age vs. share of non-liquid investments for wealth quartiles. The results from Campanale et al. with high transaction costs(TC) are used for comparison. In our model, there are no transfers between non-liquid and liquid assets before retirement, so high transaction cost results are relatively compatible with our model.

Occupation	Quart 1.	Quart 2.	Quart 3.	Quart 4.
Arts–Design–Entertainment–Sports–Media				
20–30	0.681	0.701	0.705	0.754
30–40	0.702	0.769	0.803	0.849
40–50	0.696	0.745	0.805	0.863
50–60	0.721	0.771	0.828	0.871
60–70	0.539	0.752	0.833	0.863
Business and Financial Operations				
20–30	0.626	0.648	0.615	0.632
30–40	0.653	0.671	0.712	0.774
40–50	0.659	0.700	0.793	0.848
50–60	0.658	0.692	0.775	0.848
60–70	0.547	0.695	0.759	0.841
Farming–Fishing–Forestry				
20–30	0.774	0.810	0.856	0.901
30–40	0.772	0.893	0.946	0.965
40–50	0.758	0.854	0.938	0.965
50–60	0.751	0.843	0.922	0.963
60–70	0.547	0.799	0.907	0.959
Healthcare Practitioners and Technical				
20–30	0.644	0.655	0.653	0.680
30–40	0.674	0.674	0.714	0.793
40–50	0.674	0.716	0.774	0.832
50–60	0.692	0.738	0.793	0.851
60–70	0.663	0.746	0.797	0.857
Legal				
20–30	0.644	0.614	0.607	0.632
30–40	0.659	0.649	0.717	0.802
40–50	0.664	0.709	0.760	0.827
50–60	0.670	0.675	0.730	0.828
60–70	0.630	0.699	0.791	0.859
Production				
20–30	0.667	0.704	0.751	0.826
30–40	0.682	0.770	0.843	0.900
40–50	0.694	0.779	0.849	0.909
50–60	0.684	0.771	0.858	0.912
60–70	0.589	0.757	0.839	0.904
All Occupations				
20–30	0.680	0.723	0.743	0.735
30–40	0.707	0.769	0.806	0.830
40–50	0.708	0.774	0.819	0.847
50–60	0.703	0.774	0.817	0.856
60–70	0.610	0.755	0.815	0.860
Campanale et al. TC high				
20–30	0.077	0.471	0.467	0.577
30–40	0.575	0.591	0.547	0.739
40–50	0.539	0.621	0.757	0.704
50–60	0.70	0.765	0.791	0.698
60–70	0.735	0.767	0.751	0.706
70–80	0.562	0.701	0.756	0.667

**Figure A1:**
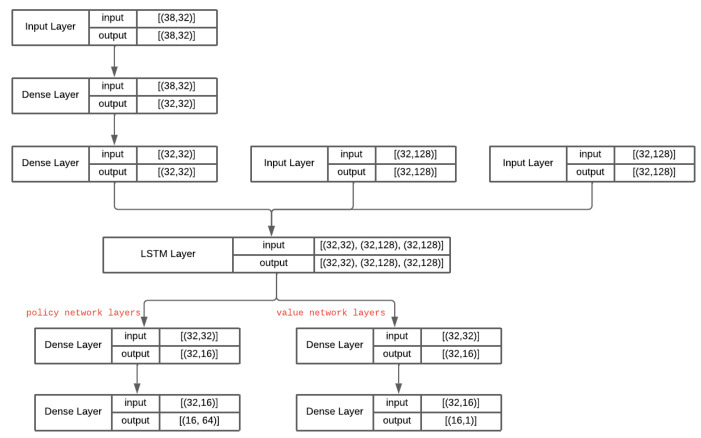
Neural Architecture.

**Figure A2:**
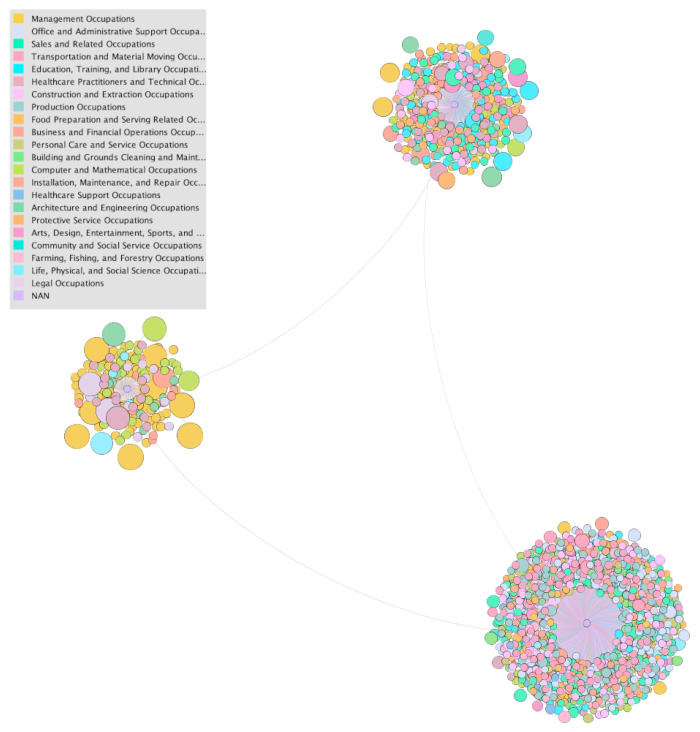
Graph plot where occupations are reflected with colours and income is reflected with the size of nodes. The graph consists of three sub-groups representing three neighbourhoods with three differing income levels as high, middle, and low income, and central nodes of each group represent the neighbourhoods.

**Figure A3:**
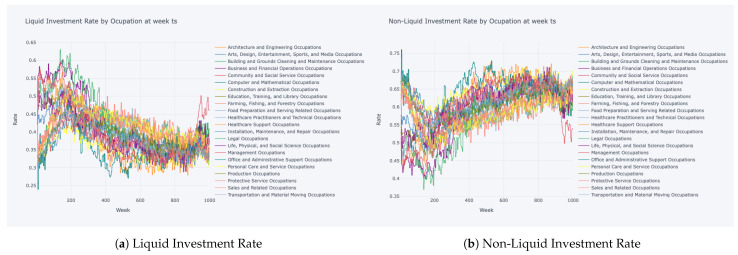
Liquid and non-liquid investment rate by occupation at week ts.

**Figure A4:**
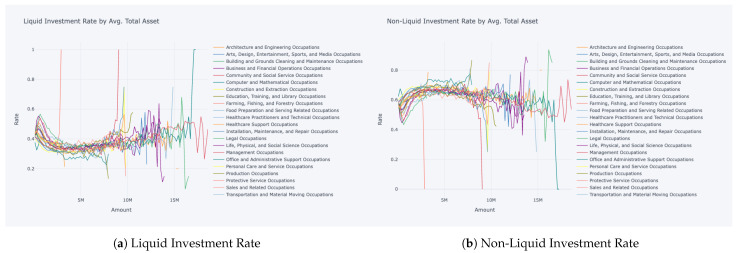
Liquid and non-liquid investment rate by OCC at amount.

**Figure A5:**
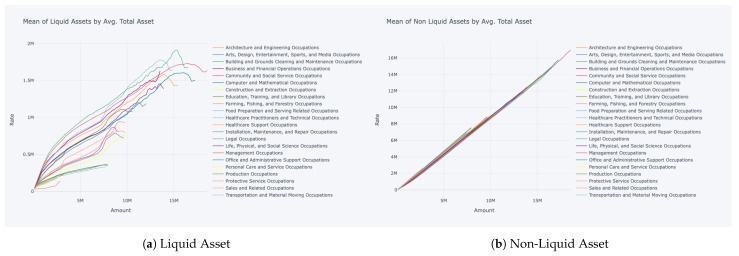
Liquid and non-liquid assets by occupation in total amount.

**Figure A6:**
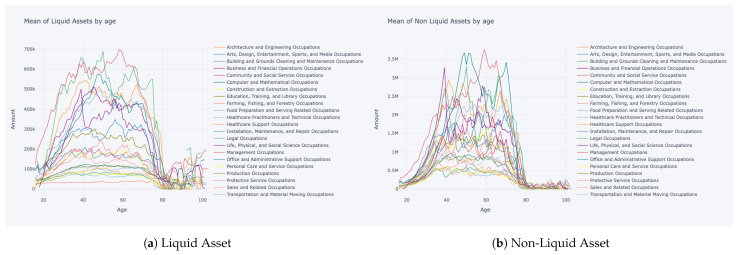
Liquid and non-liquid assets by occupation at age.

**Figure A7:**
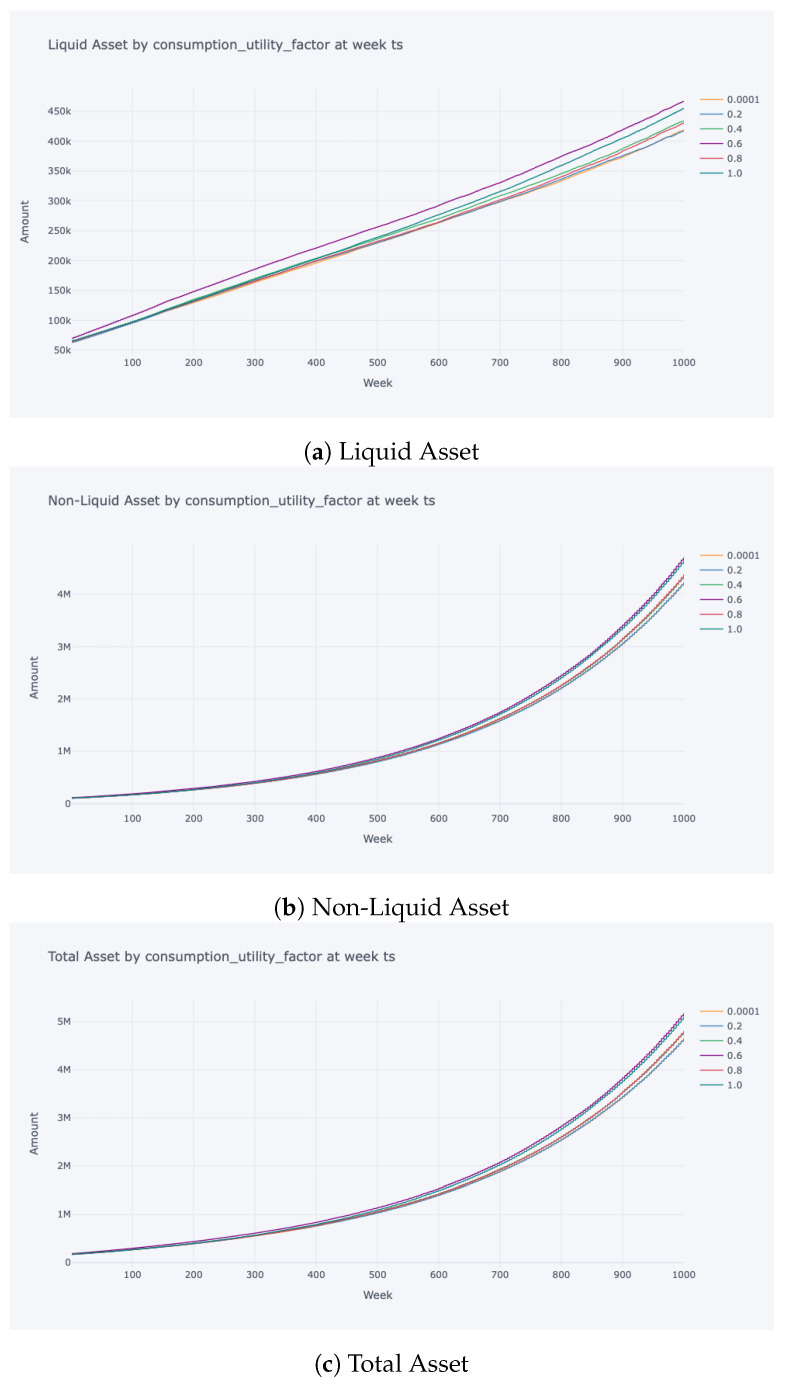
Total asset, non-liquid asset, liquid asset at week ts by consumption utility factor.

**Figure A8:**
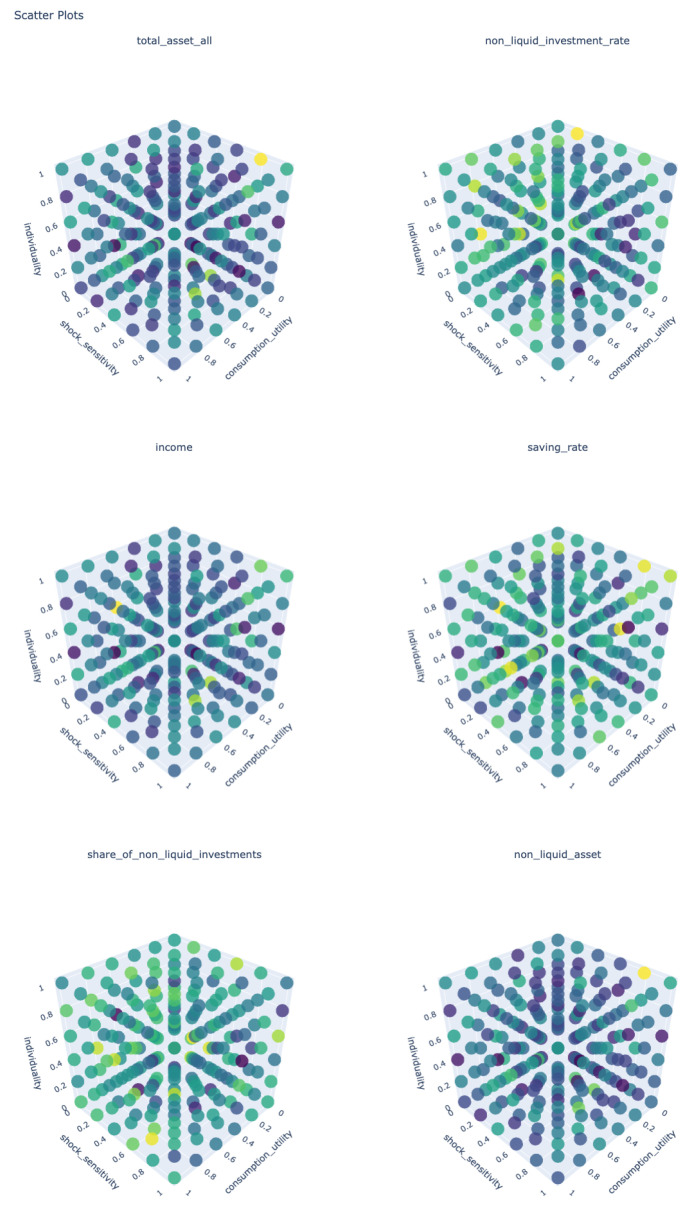
3D scatter plot of each indicator relative to the behavioural parameters of the agents, where dark blue indicates lower values and light yellow indicates higher values, which reflects how the parameters are affecting the values such as accumulated assets, investment ratess or share of non-liquid assets. The income vs. parameters plot is provided for convenience, and the income itself is not affected by the behavioural parameters.

**Figure A10:**
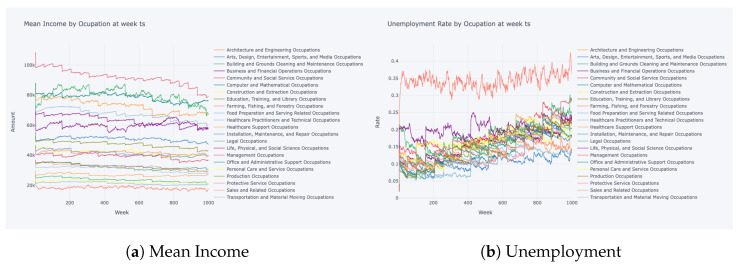
Mean Income and unemployment by occupation at week ts.

**Figure A11:**
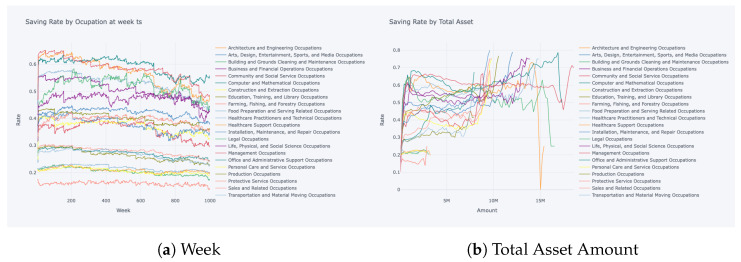
Saving rate by occupation at week ts and saving rate by occupation at amount capped at 10 M.

**Figure A12:**
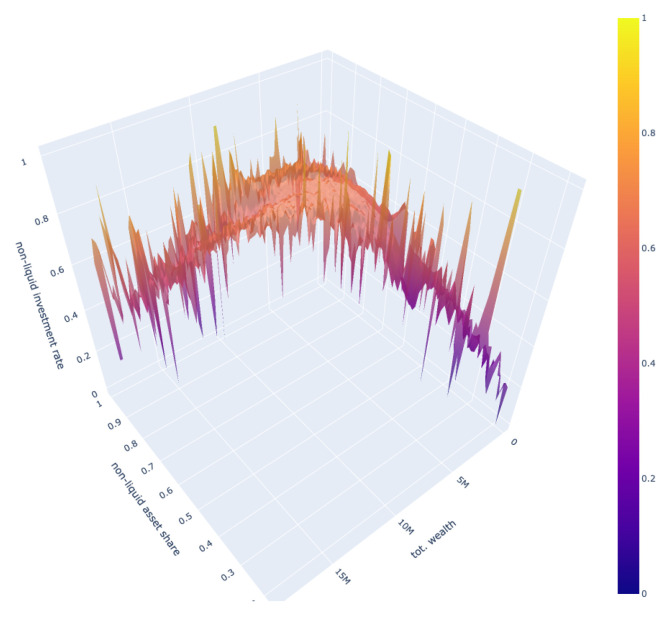
3D surface plot of share of non-liquid assets in x-axis with respect to total asset wealth in y-axis and corresponding decision of non-liquid asset investment rate in z-axis.

## Data Availability

The data that are used as input for calibration of our simulation and model can be found at [29,30]. The resulting tables that we generate can be found in the manuscript and appendices.

## Appendix A. Model Card

**Table A1:** Parameters.

Simulation Parameters	
Parallel Environment Count	32
Income Calibration Data	USA CPS 2019 Median weekly earnings
Unemployment Duration Data	USA CPS 2019 Unemployment duration table
use_min_max_scaler	1
time steps	1000
consumption_crisis_penalty	100,000
invalid_action_penalty_modifier	1000
retirement_age	65
retirement_salary_multiplier	0.8
death_rate	USA SSA Actuarial Life Table
Agent States	”OCC_CODE”, “age”, “income”, “consumption_utility_factor”, “shock_sensitivity_factor”,“individuality_factor”, “non_liquid_asset”, “liquid_asset”]
**Market Parameters**	
monthly_market_interest_rate	0
CPI	0
monthly_non_liquid_asset_return_rate	0.0125
monthly_liquid_asset_return_rate	0.0025
monthly_minimum_consumption	1073 (2021 USA Poverty Guidelines)
monthly_minimum_wage	1160
ML Parameters	
batch_size	14,656
$c_{1}$	0.5
$c_{2}$	0.01
*e*	1 × 10${}^{-5}$
γ	0.99
λ	0.95
n_lstm	128
n_steps	1

## Appendix D. Discussion Regarding the Previous Work and Limitations of the Model

The key contributions of previous work in the field of dynamic optimisation for retirement planning include the development of life-cycle models of income, consumption, and portfolio allocation, as well as the incorporation of behavioural parameters and the consideration of individual differences in decision-making. Previous work has also highlighted the importance of incorporating age-dependent income dynamics and the potential for income shocks in order to capture the complexities of real-world income trajectories.

Overall, while previous work has made important contributions to the field of retirement planning, there is still a need for more realistic and flexible models that can capture the unique income profiles and decision-making styles of individual individuals. The proposed deep reinforcement learning model addresses some of these limitations by allowing for the incorporation of individual behavioural parameters and by providing a dynamic optimisation approach that can adapt to changes in income and investment options over time. The key assumptions of the proposed deep reinforcement learning model include the assumptions that agents have access to a range of different investment options and that they are able to switch between these options depending on their current income and the expected future income. The model also assumes that agents have access to accurate and up-to-date information about their current income and the expected future income as well as information about the different investment options and their potential returns.

One of the main limitations of the proposed model is that it relies on the availability of accurate and comprehensive data about individual income profiles and investment options. Without access to high-quality data, the model may not be able to accurately capture the unique income profiles and decision-making styles of individual individuals. Another limitation of the model is that it assumes that agents are able to make rational and optimal decisions about their saving and investment strategies, which may not always be the case in the real world.

## Appendix E. Cross-Sectional Analysis

The Figure A4 reflects the relationship between liquid investment rate and wealth for amounts less than USD 5M, and three distinctive behaviours are observable; one is Computer and Mathematical Occupations, which starts at the lowest liquid investment rate, and the other group represents the majority of the occupations representing most of low- and mid-income occupations, which start at nearly 35% but then lower their liquid investment rates when the total wealth increases. The third group consist mostly of the high income occupations, such as Management Occupations, which increase their liquid investment rate with total asset increase until nearly USD 500K, and at that point they start to decrease their liquid investment rate.

Figure A5 shows that the increase in liquid assets slows with increasing total wealth, which reflects the fact that the need for security buffer savings decreases and the reward of non-liquid assets is higher. On the contrary, the increase in non-liquid assets with respect to the total wealth increase speeds up at higher amounts and converges to a stable linear trajectory.

The distribution of assets with respect to age in Figure 5 highly differentiates according to the occupation. For example, Management and Legal Occupations have the highest value of assets while Farming, Fisheries, and Food Preparation Occupations have the lowest level of assets. Asset differentiation with respect to age depends heavily on the occupation type, some occupations show great variations for the income asset values while other occupations provide minimal savings opportunity due to the income being merely sufficing to finance consumption.

## Appendix F. Effects of Behavioural Parameters

In order to further refine the behavioural parameterisation of agents in the proposed deep reinforcement learning model, we introduced three additional factors: consumption utility, individuality, and shock sensitivity. These factors capture additional aspects of individual decision-making styles and allow for even more personalised and realistic recommendations for saving and investment decisions.

The consumption utility factor captures an individual’s preference for immediate consumption versus future savings. This factor is similar to time preference, but it takes into account not only the individual’s focus on the present or the future but also their overall utility or enjoyment from consuming goods and services. Individuals with a high consumption utility value are more focused on enjoying the present and tend to prioritise immediate consumption over long-term savings, while individuals with a low consumption utility value are more focused on the future and tend to prioritise long-term savings over immediate consumption.

The individuality factor captures an individual’s willingness to deviate from the average or typical behaviour of their peers. Individuals with a high individuality value are more likely to make unique or unconventional decisions, while individuals with a low individuality value are more likely to conform to the average or typical behaviour of their peers. This factor allows the model to capture the diversity of individual decision-making styles and to account for individuals who may be more likely to take risks or to make unconventional investment decisions.

The shock sensitivity factor captures an individual’s sensitivity to sudden negative changes or shocks to their income. Individuals with a high shock sensitivity value are more likely to be affected by income shocks and may be more conservative in their investment decisions as a result, while individuals with a low shock sensitivity value are less likely to be affected by income shocks and may be more willing to take on risky investments. This factor allows the model to capture the effects of income volatility on individual decision-making and to provide more personalised recommendations for saving and investment decisions in the face of income shocks.

Incorporating these three additional factors into the behavioural parameterisation of agents allows the proposed deep reinforcement learning model to capture a wider range of individual decision-making styles and to provide even more personalised and realistic recommendations for saving and investment decisions. This allows the model to better reflect the diversity and complexity of individual preferences and to provide more tailored and effective recommendations for retirement planning.

These factors capture the behaviour of agents, and they impact how agents perceive, understand, and act in their environment. The consumption utility factor is necessary for quantifying how agents value immediate consumption, which can be interpreted as level of consumerism, or temporal preference and eagerness. The shock sensitivity factor is a parameter helpful for capturing the agent’s perception of the consumption change, which can amplify the effects of the changes and force agents to avoid abrupt changes, and an alternative interpretation can be as risk aversion modifier that augments the utility. The individuality factor models the level of influence an agent’s social network exerts on the agent. This is achieved by factoring in the information transmitted from the neighbourhood. The increase in the liquid assets reflect a linear increase; on the contrary, the increase in non-liquid assets is exponential due to interest income of the assets. The distribution of outcomes reflect heterogeneous characteristics according to behavioural parameters and the relationship between parameters and outcomes are non-linear.

## Appendix G. Tables

**Table A2:** Occupation vs. rates: the saving rate denotes to the average monthly saving rate of the members of each occupation, and the non-liquid investment rate denotes the average of the decided rate of allocating monthly savings to non-liquid investments for each occupation, the share of non-liquid investments denotes the share of non-liquid assets with respect to all of the investments averaged for each occupation.

Occupation Title	Saving Rate	Non Liquid Investment Rate	Share of Non Liquid Investments
Architecture and Engineering	0.578	0.607	0.730
Arts, Design, Entertainment, Sports, and Media	0.422	0.587	0.756
Building and Grounds Cleaning and Maintenance	0.207	0.576	0.777
Business and Financial Operations	0.512	0.557	0.714
Community and Social Service	0.363	0.623	0.773
Computer and Mathematical	0.596	0.643	0.769
Construction and Extraction	0.374	0.649	0.787
Education, Training, and Library	0.404	0.574	0.743
Farming, Fishing, and Forestry	0.160	0.569	0.845
Food Preparation and Serving Related	0.218	0.574	0.790
Healthcare Practitioners and Technical	0.533	0.576	0.737
Healthcare Support	0.210	0.576	0.777
Installation, Maintenance, and Repair	0.381	0.635	0.786
Legal	0.519	0.550	0.703
Life, Physical, and Social Sciences	0.471	0.603	0.732
Management	0.590	0.584	0.722
Office and Administrative Support	0.274	0.608	0.778
Personal Care and Service	0.208	0.573	0.778
Production	0.267	0.608	0.782
Protective Service	0.396	0.622	0.777
Sales and Related	0.282	0.578	0.767
Transportation and Material Moving	0.269	0.613	0.789

**Table A3:** Age vs. rates: Consumption rates are defined as consumption amount divided by income. Consumption rates are compared to the literature by extracting values from plots of [10]; their research differs by our work such that the income values exclude contributions toward pension income, and savings are used as a mean to finance consumption deficit, especially during retirement. So, during retirement, there are positive consumption rates, which means that the pension deficit is financed by spending savings. This definition difference causes consumption rates to be much higher.

Age	Non-Liquid Investment Rate	Share of Non-Liquid Investments	Consumption Rate	Cocco et al. Consumption Rate
21	0.551	0.677	0.648	0.884
22	0.546	0.684	0.634	0.915
23	0.553	0.690	0.629	0.948
24	0.554	0.701	0.628	0.976
25	0.556	0.710	0.623	0.996
26	0.558	0.717	0.611	0.998
27	0.562	0.728	0.608	0.999
28	0.567	0.736	0.607	0.999
29	0.571	0.743	0.609	0.998
30	0.572	0.750	0.606	0.996
31	0.579	0.756	0.598	0.987
32	0.584	0.764	0.595	0.979
33	0.587	0.771	0.596	0.972
34	0.590	0.777	0.591	0.966
35	0.592	0.781	0.591	0.962
36	0.594	0.782	0.588	0.960
37	0.596	0.783	0.583	0.959
38	0.597	0.786	0.578	0.959
39	0.597	0.786	0.575	0.960
40	0.600	0.789	0.571	0.962
41	0.597	0.787	0.563	0.963
42	0.601	0.785	0.562	0.965
43	0.603	0.785	0.561	0.966
44	0.603	0.787	0.562	0.966
45	0.604	0.786	0.559	0.966
46	0.605	0.788	0.561	0.966
47	0.604	0.787	0.559	0.965
48	0.605	0.789	0.557	0.964
49	0.603	0.788	0.555	0.963
50	0.605	0.788	0.555	0.963
51	0.609	0.789	0.557	0.964
52	0.607	0.787	0.558	0.966
53	0.607	0.785	0.564	0.970
54	0.605	0.785	0.563	0.976
55	0.606	0.786	0.568	0.985
56	0.604	0.787	0.569	0.996
57	0.604	0.787	0.569	1.011
58	0.604	0.790	0.573	1.029
59	0.606	0.790	0.572	1.051
60	0.606	0.789	0.578	1.077
61	0.609	0.788	0.578	1.107
62	0.606	0.788	0.572	1.142
63	0.607	0.784	0.572	1.180
64	0.606	0.785	0.570	1.223

**Table A4:** Saving Rate by Occupation and Age.

Occupation	20–30	30–40	40–50	50–60
Architecture and Engineering	0.669	0.68	0.658	0.668
Arts, Design, Entertainment, Sports, and Media	0.465	0.448	0.445	0.484
Building and Grounds Cleaning and Maintenance	0.237	0.237	0.236	0.235
Business and Financial Operations	0.556	0.602	0.594	0.599
Community and Social Service	0.447	0.422	0.433	0.41
Computer and Mathematical	0.639	0.672	0.671	0.674
Construction and Extraction	0.419	0.435	0.432	0.42
Education, Training, and Library	0.456	0.471	0.472	0.436
Farming, Fishing, and Forestry	0.181	0.18	0.176	0.184
Food Preparation and Serving Related	0.236	0.235	0.235	0.236
Healthcare Practitioners and Technical	0.598	0.615	0.591	0.578
Healthcare Support	0.236	0.235	0.234	0.234
Installation, Maintenance, and Repair	0.446	0.427	0.434	0.412
Legal	0.603	0.584	0.592	0.573
Life, Physical, and Social Sciences	0.56	0.549	0.556	0.535
Management	0.683	0.692	0.692	0.68
Office and Administrative Support	0.31	0.315	0.317	0.311
Personal Care and Service	0.238	0.236	0.237	0.236
Production	0.309	0.305	0.31	0.303
Protective Service	0.429	0.437	0.445	0.426
Sales and Related	0.334	0.313	0.323	0.312
Transportation and Material Moving	0.307	0.302	0.315	0.298

## References

[1] OECD (2020). Pension Markets in Focus 2020. www.oecd.org/finance/pensionmarketsinfocus.htm.

[2] ONS (2019). Occupational Pension Schemes in the UK. https://www.ons.gov.uk/peoplepopulationandcommunity/personalandhouseholdfinances/pensionssavingsandinvestments/datasets/occupationalpensionschemessurvey.

[3] Wilkinson L., Adams J. (2021). What impact has the COVID-19 pandemic had on underpensioned groups?. Pensions Policy Inst..

[4] Abraham K., Haltiwanger J., Sandusky K., Spletzer J. (2017). Measuring the gig economy: Current knowledge and open issues. Measuring and Accounting for Innovation in the 21st Century.

[5] Ozhamaratli F., Kitov O., Barucca P. (2022). A generative model for age and income distribution. EPJ Data Sci..

[6] Ando A., Modigliani F. (1963). The “Life Cycle” Hypothesis of Saving: Aggregate Implications and Tests. Am. Econ. Rev..

[7] Samuelson P.A. (1969). Lifetime Portfolio Selection By Dynamic Stochastic Programming. Rev. Econ. Stat..

[8] Merton R.C. (1969). Lifetime Portfolio Selection under Uncertainty: The Continuous-Time Case. Rev. Econ. Stat..

[9] Merton R.C. (1971). Optimum consumption and portfolio rules in a continuous-time model. J. Econ. Theory.

[10] Cocco J.F., Gomes F.J., Maenhout P.J. (2005). Consumption and Portfolio Choice over the Life Cycle. Rev. Financ. Stud..

[11] Campanale C., Fugazza C., Gomes F. (2015). Life-cycle portfolio choice with liquid and illiquid financial assets. J. Monet. Econ..

[12] Epstein L.G., Zin S.E. (1989). Substitution, Risk Aversion, and the Temporal Behavior of Consumption and Asset Returns: A Theoretical Framework. Econometrica.

[13] Dahlquist M., Setty O., Vestman R. (2016). On the Asset Allocation of a Default Pension Fund. Ssrn Electron. J..

[14] Zheng S., Trott A., Srinivasa S., Naik N., Gruesbeck M., Parkes D.C., Socher R. (2020). The AI Economist: Improving Equality and Productivity with AI-Driven Tax Policies. arXiv.

[15] Gomes F.J., Michaelides A. (2002). Life-Cycle Asset Allocation: A Model with Borrowing Constraints, Uninsurable Labor Income Risk and Stock-Market Participation Costs. Ssrn Electron. J..

[16] Acemoglu D., Ozdaglar A., Tahbaz-Salehi A. (2015). Systemic risk and stability in financial networks. Am. Econ. Rev..

[17] Barberis N.C. (2013). Thirty years of prospect theory in economics: A review and assessment. J. Econ. Perspect..

[18] Pratt J.W. (1964). Risk Aversion in the Small and in the Large. Econometrica.

[19] Konda V., Tsitsiklis J. (1999). Actor-critic algorithms. Adv. Neural Inf. Process. Syst. 1008–1014.

[20] Hill A., Raffin A., Ernestus M., Gleave A., Kanervisto A., Traore R., Dhariwal P., Hesse C., Klimov O., Nichol A. (2018). Stable Baselines. https://github.com/hill-a/stable-baselines.

[21] Schulman J., Wolski F., Dhariwal P., Radford A., Klimov O. (2017). Proximal Policy Optimization Algorithms. arXiv.

[22] Schulman J., Moritz P., Levine S., Jordan M., Abbeel P. (2015). High-Dimensional Continuous Control Using Generalized Advantage Estimation. arXiv.

[23] Huang S., Dossa R.F.J., Raffin A., Kanervisto A., Wang W. (2022). The 37 Implementation Details of Proximal Policy Optimization. ICLR Blog Track.

[24] Williams R.J., Peng J. (1991). Function Optimization using Connectionist Reinforcement Learning Algorithms. Connect. Sci..

[25] Kingma D.P., Ba J. (2014). Adam: A method for stochastic optimization. arXiv.

[26] Terry J.K., Black B., Grammel N., Jayakumar M., Hari A., Sulivan R., Santos L., Perez R., Horsch C., Dieffendahl C. (2020). PettingZoo: Gym for Multi-Agent Reinforcement Learning. arXiv.

[27] Hochreiter S., Schmidhuber J. (1997). Long Short-Term Memory. Neural Comput..

[28] Cane J., O’Connor D., Michie S. (2012). Validation of the theoretical domains framework for use in behaviour change and implementation research. Implement. Sci..

[29] BLS (2019). 2019 Annual Averages—Household Data—Tables from Employment and Earnings.

[30] Department of Health and Human Services (2019). Annual Update of the HHS Poverty Guidelines.

[31] SSA (2017). Actuarial Life Table—SSA.

